# Comparative Study on Topological Properties of the Whole-Brain Functional Connectome in Idiopathic Rapid Eye Movement Sleep Behavior Disorder and Parkinson’s Disease Without RBD

**DOI:** 10.3389/fnagi.2022.820479

**Published:** 2022-04-11

**Authors:** Shuai Chen, Sheng-Hui Wang, Ying-Ying Bai, Jie-Wen Zhang, Hong-Ju Zhang

**Affiliations:** Department of Neurology, Zhengzhou University People’s Hospital, Henan Provincial People’s Hospital, Zhengzhou, China

**Keywords:** functional connectome, iRBD, graph theory, Parkinson’s disease, cognition

## Abstract

**Purpose:**

Idiopathic rapid eye movement Sleep Behavior Disorder (iRBD) is considered as a prodromal and most valuable warning symptom for Parkinson’s disease (PD). Although iRBD and PD without RBD (nRBD-PD) are both α-synucleinopathies, whether they share the same neurodegeneration process is not clear enough. In this study, the pattern and extent of neurodegeneration were investigated and compared between early-stage nRBD-PD and iRBD from the perspective of whole-brain functional network changes.

**Methods:**

Twenty-one patients with iRBD, 23 patients with early-stage nRBD-PD, and 22 matched healthy controls (HCs) were enrolled. Functional networks were constructed using resting-state functional MRI (fMRI) data. Network topological properties were analyzed and compared among groups by graph theory approaches. Correlation analyses were performed between network topological properties and cognition in the iRBD and nRBD-PD groups.

**Results:**

Both patients with iRBD and patients with early-stage nRBD-PD had attention, executive function, and some memory deficits. On global topological organization, iRBD and nRBD-PD groups still presented small-worldness, but both groups exhibited decreased global/local efficiency and increased characteristic path length. On regional topological organization, compared with HC, nRBD-PD presented decreased nodal efficiency, decreased degree centrality, and increased nodal shortest path length, while iRBD presented decreased nodal efficiency and nodal shortest path. For iRBD, brain regions with decreased nodal efficiency were included in the corresponding regions of nRBD-PD. Nodal shortest path changes were significantly different in terms of brain regions and directions between nRBD-PD and iRBD. Attention deficits were correlated with local topological properties of the occipital lobe in both iRBD and nRBD-PD groups.

**Conclusion:**

Both global and local efficiency of functional networks declined in nRBD-PD and iRBD groups. The overlaps and differences in local topological properties between nRBD-PD and iRBD indicate that iRBD not only shares functional changes of PD but also presents distinct features.

## Introduction

Idiopathic rapid eye movement sleep behavior disorder (iRBD) is characterized by dream enactment and increased muscle tone during the rapid eye movement (REM) sleep period. Longitudinal studies have found that the probability of iRBD converting to α-synucleinopathy [i.e., Parkinson’s disease (PD), Lewy body dementia, multiple system atrophy (MSA)] at 10 years after onset is 80∼90% (Galbiati et al., 2019; Postuma et al., 2019). Therefore, iRBD is considered as the most valuable warning symptom for PD. Although attributed to lower brainstem dysfunction, there were considerable clinical, imaging, and pathological evidence of wide neurodegeneration in the striatum, substantia nigra, limbic system, and cortex for iRBD. Clinically, patients with iRBD could present some non-motor symptoms similar to PD, such as cognitive impairment, hyposmia, and autonomic dysfunction (Hu, 2020). Dopamine transporter (DAT) imaging revealed decreased dopaminergic uptake in the nigrostriatal system in patients with iRBD (Bauckneht et al., 2018). Resting-state functional MRI (rs-fMRI) studies on iRBD found functional connectivity changes within the basal ganglia, cortico-striatal, and cortico-cortical networks (Campabadal et al., 2021; Zhang et al., 2021).

Although iRBD exhibits many features similar to PD, iRBD is not simply a prodromal PD. There were a number of studies that comparatively investigated the clinical presentations in PD with (RBD-PD) or without RBD (nRBD-PD). Compared with nRBD-PD, RBD-PD presents distinct clinical features, such as severe motor symptoms, severe cognitive impairment, and more rapid progression (Pagano et al., 2018). Although iRBD and nRBD-PD are both α-synucleinopathies, whether they share the same neurodegeneration process is not clear enough. The long-term phenoconversion for patients with iRBD is heterogeneous. Thus, the neurodegeneration basis of iRBD may also be heterogeneous. However, histopathological studies are not practical for iRBD. This study aimed to explore and compare the distributions of neurodegeneration in patients with iRBD and PD from the perspective of fMRI. Particularly, whether iRBD presents with some functional changes that are not presented in PD. To maximally reveal this difference, the current comparative study enrolled patients with iRBD and patients with early-stage PD without RBD.

The human brain network has the properties of functional integration and functional segregation to achieve high efficiency of information transfer at a low cost. rs-fMRI is a non-invasive method that uses blood oxygenation level-dependent fMRI (BOLD-fMRI) to investigate spontaneous brain activity and construct functional connectivity. Both seed-based analysis and independent component analysis (ICA) could only characterize local networks of brain connectivity. Graph theoretical analysis has the advantage of characterizing the global and local topological properties of brain functional networks (Medaglia, 2017). Moreover, graph theory can interpret the cognitive impairment in neurodegenerative diseases from the perspective of information transfer efficiency.

To date, there were few graph theory resting-state MRI studies on iRBD. One study found decreased functional connectivity in the posterior cortex and decreased centrality of the superior parietal lobule in patients with iRBD. In addition, functional connectivity between the superior parietal lobule and inferior temporal gyrus was correlated with mental processing speed (Campabadal et al., 2020). In another study, the network organizations were reconstructed with the limbic lobe as the largest central node in patients with PD and concomitant RBD (Li et al., 2020).

In this study, topological properties of the whole-brain functional network were analyzed by graph theory in patients with iRBD and nRBD-PD. We hypothesize that the topological properties of iRBD do not merely reflect prodromal PD, but also have unique features.

## Materials and Methods

### Participants

Twenty-one patients with iRBD and 23 patients with PD from the Department of Neurology, People’s Hospital of Zhengzhou University were included in this study. All patients with iRBD underwent polysomnography (PSG), and the diagnosis was made according to the criteria from the third edition of the International Classification of Sleep Disorders (ICSD-3). The diagnosis of PD met the movement disorder society’s (MDS) PD criteria. All patients with PD had no concomitant RBD. nRBD-PD was confirmed by the RBD screening questionnaire (RBDSQ) and PSG. That is, the PD subject that fulfilled both an RBDSQ score of less than 5 points and a negative PSG examination could be classified as nRBD-PD. Twenty-two sex and age-matched healthy controls (HCs) came from the health checkup center of the hospital. None of the subjects had the following conditions: (1) Cognitive impairment [with the Mini Mental State Examination (MMSE) score < 25 points]; (2) Secondary RBD; (3) Anxiety and depression disorders; (4) Other sleep disorders; (5) Other central nervous system diseases; (6) Drug or alcohol addiction; (7) Severe heart, lung, liver, kidney, and endocrine diseases; and/or (8) Deafness and MRI contraindications.

This study was approved by the Medical Ethics Committee of People’s Hospital of Zhengzhou University (No:201705). Informed consent was obtained from all subjects.

### Neuropsychological and Clinical Assessment

All subjects underwent neuropsychological and motor evaluations. Global cognitive function was assessed by MMSE. Attention function was assessed by the Digit Ordering Test (DOT) and the Trail Making Test A (TMT-A) (Hoppe et al., 2000; Zhao et al., 2013), executive function by the Trail Making Test B (TMT-B) and the Symbol Digital Modalities Test (SDMT) (Zhao et al., 2013), visuospatial ability by the Rey-Osterrieth Complex Figure Test (ROCFT-copy), and memory by Auditory Verbal Learning Test-Huashan (AVLT-H) (Guo et al., 2009). In AVLT-H, short-term delayed free recall (SR) was tested 5 min after three rounds of the learning and recalling phase, while delayed recall (DR) was tested 30 min after three rounds of the learning and recalling phase (Guo et al., 2009). Motor function was assessed by Hoehn and Yahr (H&Y) staging and Movement Disorder Society Unified Parkinson’s Disease Rating Scale part III (MDS-UPDRS-III).

### MRI Acquisition and Preprocessing

All subjects underwent brain MRI (GE discovery Mr. 750 3.0 T, United States) at the imaging center of People’s Hospital of Zhengzhou University. Sponge pads were used to stabilize subjects’ heads and were provided with earplugs during the scan. They were told to be quiet, keep their eyes closed, and think of nothing. The subjects were instructed not to fall asleep before the scan and were asked whether they fell asleep after the scan to ensure all data met the requirements. T1-weighted MRI was obtained by the 3D fast spoiled gradient recalled sequence (3D-FSPGR) at the magnetic field strength of 3.0T with the following parameters: repetition time (TR) = 8.2 ms, echo time (TE) = 3.22 ms, inversion time (TI) = 450 ms, matrix = 256 × 256, slice thickness = 1.0 mm, slices = 156, field of view (FOV) = 240 mm, and flip angle (FA) = 12°. rs-fMRI data were acquired using a standard gradient-recalled echo-echo planar imaging (GRE-EPI) sequence with the following parameters: TR = 2,000 ms, TE = 30 ms, TI = 450 ms, FOV = 240 mm, FA (flip angle) = 90°, matrix size = 64 × 64, slices = 39, and slice thickness = 4 mm. Each functional run contained 210 image volumes in a total imaging time of 410 s.

Data Processing Assistant for rs-fMRI (DPARSF) software^1^ was used for data preprocessing. To eliminate the effect of magnetic saturation at the beginning of the scan, the first 10 volumes of rs-fMRI data were discarded. After slice-time correction and head motion correction, none of the subjects had head displacements that exceeded 3 mm or 3.0° for translation or rotational parameters. The images were then spatially normalized to the Montreal Neurological Institute (MNI) space and smoothed with a 4 mm full width at half maximum (FWHM) Gaussian kernel. A temporal bandpass filter (0.01–0.08 Hz) was performed to eliminate high-frequency physiological noise and low-frequency drift. Finally, the covariates (including head motion parameters, whole-brain signals, white matter signals, and cerebrospinal fluid signals) were regressed out.

### Network Construction and Graph Theory Analysis

Network construction: Connectivity analysis was based on an automated anatomical labeling (ALL) template which divided the whole brain into 90 brain regions (Tzourio-Mazoyer et al., 2002). Each region was regarded as a node of the brain network, and the interaction between each two brain regions was regarded as an edge of the brain network. Time series of the 90 brain regions of each subject were extracted. Pearson correlation coefficients were calculated to obtain a 90 × 90 correlation matrix for each subject.

Graph theory analysis: In the Matlab environment, GRETNA^2^ software was used to obtain the topological properties of the brain function network by graph theory. A weighted connection matrix was generated for each participant with positive correlations retained for network analysis. We applied the sparsity threshold S to select all connection matrices ranging from *S* = 0.05 to 0.5 with an interval of 0.05 (Qiu et al., 2021). The area under the curve (AUC) value was calculated for each network property among all the sparsity. Global properties included small-worldness, global efficiency, and local efficiency. Local properties include nodal degree centrality, nodal efficiency, and nodal shortest path length (Lp). The characteristic Lp refers to the average shortest Lps between all pairs of nodes, reflecting the overall efficiency of information transfer between different brain regions. A smaller characteristic Lp indicates a faster information transfer speed of the network. The clustering coefficient (C) indicates the information processing efficiency within local brain regions. In the definition of small-world properties, normalized clustering coefficient γ = C_real/C_random, normalized characteristic path length λ = L_real/L_random, and small-world coefficient σ = γ/λ, the small-worldness is satisfied if σ > 1; γ > 1 and λ≈ 1. Global efficiency also reflects the global information transfer in a way that is contrary to characteristic path length. Local efficiency is similar to the clustering coefficient, which reflects the efficiency of local information transfer in the network. Local properties include nodal degree centrality, nodal efficiency, and nodal shortest path length. Degree centrality is defined as the number of nodes directly connected to a given node. Nodal efficiency characterizes the efficiency of parallel information transfer of one node in the network (Rubinov and Sporns, 2010).

### Statistics Analysis

SPSS 19.0 software was used to analyze the clinical data of the iRBD group, nRBD-PD group, and HC group. Normally distributed continuous data were expressed as mean ± standard deviation (SD). One-way ANOVA was used for intra-group comparisons, and Bonferroni correction was used for *post hoc* inter-group comparisons. Non-normally distributed continuous data were expressed as median and quartile [M(P25, P75)], with the Kruskal–Wallis test for intra-group comparisons and the Mann–Whitney U test for inter-group comparisons. On GRETNA software, one-way ANOVA was used for intra-group comparisons and *t*-test for inter-group comparisons. The results were corrected by false discovery rate (FDR). Partial correlation analysis (adjusting for gender, age, and education level) was performed between local properties and raw cognitive test scores in the iRBD and nRBD-PD groups, respectively. Significance was established at *p* < 0.05.

## Results

### Demographic and Clinical Comparisons

No significant differences were observed for age, gender, and education years between the nRBD-PD, iRBD, and HC groups. There were intra-group differences in SDMT, TMT-A, TMT-B, AVLTN-SR, and MDS-UPDRS-III scores among the three groups (*p* < 0.05). Compared with the HC group, the iRBD group had lower SDMT and higher TMT-A and TMT-B scores. Compared with the HC group, the nRBD-PD group had higher TMT-A, TMT-B, and UPDRS-III scores, but lower DOT, SDMT, AVLT-DR, and AVLT-SR scores (*p* < 0.05). Compared with the iRBD group, the nRBD-PD group had higher scores of TMT-A, TMT-B, and MDS-UPDRS-III (Table 1).

**TABLE 1:** Demographics and clinical characteristics of the subjects.

	Group						
Characteristics	RBD (*n* = 21)	PD (*n* = 23)	HC (*n* = 22)	RBD vs. PD vs. HC	RBD vs. PD	RBD vs. HC	PD vs. HC
Age(years)	61.00 ± 10.68	60.13 ± 11.22	60.27 ± 7.60	0.95	1.00	1.00	1.00
Education(years)	8.81 ± 3.40	8.22 ± 3.63	8.86 ± 3.14	0.78	1.00	1.00	1.00
Male	14	14	9	0.20	0.76	0.13	0.24
Duration [years,M(P25,P75)]	3.00(2.00, 5.00)	2.50(1.00, 3.25)	n.a.	n.a.	n.a.	n.a.	n.a.
MMSE [M(P25,P75)]	27.00(26.00,28.00)	27.00(26.00,28.00)	28.00(27.00,28.00)	0.23	0.33	0.45	0.09
DOT [M(P25,P75)]	5.00(4.00,5.00)	4.00(2.72,6.00)	6.00(5.00,6.00)	0.05	0.23	0.08	**0.00**
TMT-A [M(P25,P75)]	88.00(70.00,120.00)	106.50(86.50,148.00)	60.00(49.00,80.50)	0.00	0.04	**0.01**	**0.00**
TMT-B [M(P25,P75)]	194.00(180.00,210.00)	258.50(190.75,307.25)	112.00(99.50,173.00)	0.00	0.00	**0.00**	**0.00**
SDMT [M(P25,P75)]	23.00(21.00,31.00)	20.00(17.50,28.00)	33.00(39.5,36.00)	0.00	0.11	**0.00**	**0.00**
AVLT-SR [M(P25,P75)]	6.00(5.00,8.00)	5.00(3.00,6.00)	6.00(6.00,8.00)	0.01	0.19	0.14	**0.00**
AVLT-DR [M(P25,P75)]	7.00(3.00,7.00)	4.00(3.00,6.50)	6.00(5.00,6.00)	0.07	0.10	**0.41**	**0.00**
ROCFT_copy [M(P25,P75)]	33.00(31.00,34.00)	34.00(30.00,36.00)	35.00(33.00,36.00)	0.17	0.27	0.05	0.56
MDS-UPDRS-III [M(P25,P75)]	0.00(0.00,2.00)	28.00(17.75,33.00)	0.00(0.00,1.00)	0.00	0.00	0.00	**0.00**
H-Y stage	0.00(0.00,0.00)	2.00(1.50,2.50)	n.a.	n.a.	n.a.	n.a.	n.a.

*iRBD, idiopathic rapid eye movement sleep behavior disorder; HC, healthy controls. ROCFT, Rey-osterrieth complex figure test; AVLT, auditory verbal learning test; SDMT, symbol digit modalities test; TMT, trail making test; DOT, digit ordering test; MDS-UPDRSIII, movement disorder society unified Parkinson’s disease rating scale motor section. Data are presented as mean ± standard deviation, or median (inter-quartile range) as appropriate. One-way ANOVA for intra-group comparison of normally distributed continuous data, and Bonferroni correction for post hoc inter-group comparison. Kruskal–Wallis test for intra-group comparisons of non-normally distributed data and Mann–Whitney U test for inter-group comparisons. For the comparisons of cognitive tests, p < 0.05 are shown in bold and are chose for further partial correlation analysis.*

### Global Topological Organization of the Functional Connectome

**FIGURE 1:**
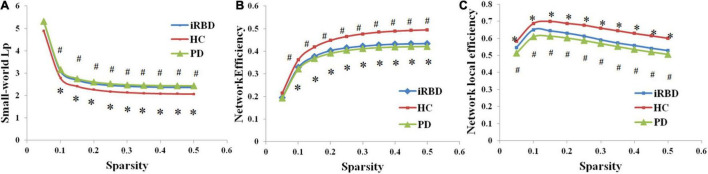
Altered global topological properties across different sparsity thresholds (0.05–0.5). **(A)** Small-world characteristic path length (Lp). **(B)** Network global efficiency. **(C)** Network local efficiency. *Indicating significant differences between idiopathic rapid eye movement (REM) sleep behavior disorder (iRBD) patients and healthy controls (HCs; *p* < 0.05). ^#^Indicating significant differences between patients with Parkinson’s disease (PD) and HCs (*p* < 0.05). iRBD, idiopathic rapid eye movement sleep behavior disorder; PD, Parkinson’s disease; HC, healthy control.

The brain networks of the iRBD group, nRBD-PD group, and HC group all presented small-worldness. However, compared with HC, decreased global/local efficiency and increased characteristic path length were observed in both iRBD and nRBD-PD groups (*p* < 0.05, FDR corrected). There were no significant differences in global topological properties between the iRBD and nRBD-PD groups (*p* > 0.05) (Figure 1).

### Regional Topological Organization of the Functional Connectome

The iRBD group, nRBD-PD group, and HC group had differences in degree centrality, nodal efficiency, and nodal shortest path length (*p* < 0.01, *p* < 0.005, *p* < 0.005, respectively, all FDR corrected). Compared with the HC group, the nRBD-PD group showed decreased degree centrality, decreased nodal efficiency, and increased nodal shortest path length. Compared with the HC group, the iRBD group showed decreased nodal efficiency but decreased nodal shortest path length. There were no differences in degree centrality, nodal efficiency, and nodal shortest path length between the iRBD and nRBD-PD groups.

**FIGURE 2:**
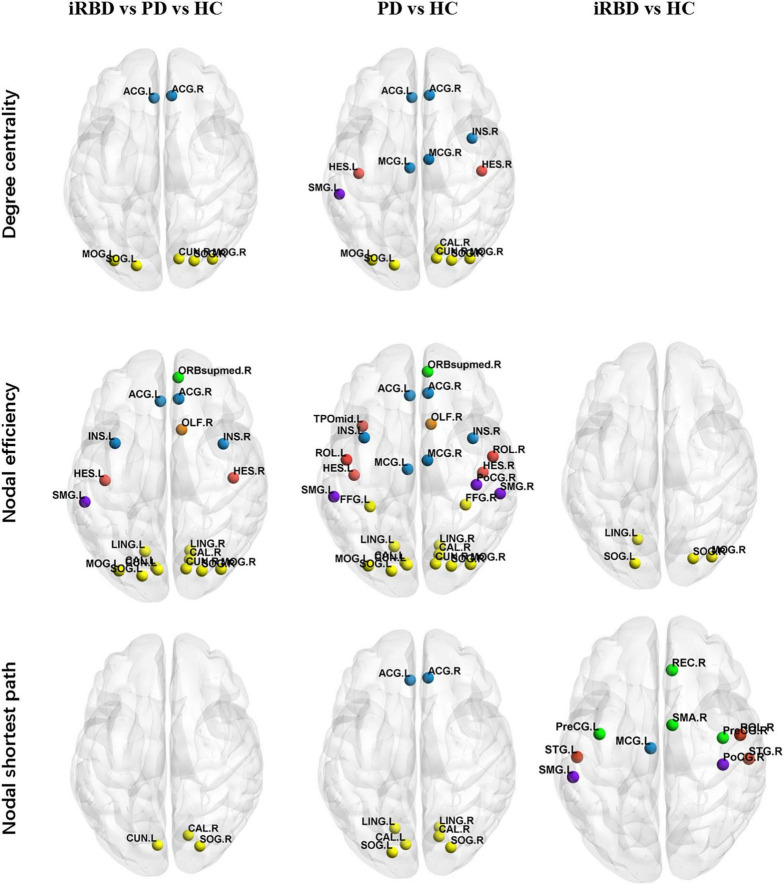
Altered local topological properties in iRBD, PD, and HC. (*p* < 0.01, *p* < 0.005, *p* < 0.005, respectively, all FDR corrected). Details are listed in Table 3. Different brain regions were indicated by colors. Yellow: occipital lobe; red: temporal lobe; green: frontal lobe; purple: parietal lobe; blue: limbic lobe; orange: olfactory bulb. ORB mid, middle frontal orbital, orbital part; INS, insula; ACG, anterior cingulum gyrus; MCG, middle cingulum gyrus; LING, lingual; SOG, superior occipital gyrus; MOG, middle occipital gyrus; IOG, inferior occipital gyrus; PoCG, postcentral gyrus; SMG, supramarginal gyrus; REC, rectus gyrus; ROL, rolandic operculum.

On nodal efficiency, nRBD-PD exhibited more altered brain regions than iRBD. The brain regions with decreased nodal efficiency in the nRBD-PD group also covered the corresponding altered regions in the iRBD group. However, opposite changes were seen on the nodal shortest path between the iRBD and the nRBD-PD group. Compared with the HC group, the iRBD group exhibited decreased nodal shortest paths in the precentral gyrus, postcentral gyrus, supramarginal gyrus, superior temporal gyrus, supramotor area, straight gyrus, middle cingulate gyrus, and Rolandic operculum. But the nRBD-PD group exhibited increased nodal shortest paths in the lingual gyrus, calcarine gyrus, superior occipital gyrus, and anterior cingulate gyrus (Figure 2 and Tables 2–4).

**TABLE 2:** Alterations of degree centrality among iRBD, PD, and HC.

	*P*-value/*T*-value			
Brain regions	RBD vs. PD vs. HC	PD vs. HC	RBD vs. HC	RBD vs. PD
INS.R	0.0115/4.8185	**0.007/−2.8467**		
ACG.L	**0.0033/6.3003**	**0.0016/−3.4028**		
ACG.R	**0.0020/6.9334**	**0.0017/−3.3743**		
MCG.L	0.0207/4.1437	**0.0034/−3.1236**		
MCG.R	0.0311/3.6843	**0.0073/−2.8333**		
CAL.R	0.0180/4.3051	**0.0023/−3.2645**		
CUN.R	**0.0025/6.6455**	**0.0008/−3.6454**		
SOG.L	**0.0011/7.6642**	**0.0011/−3.5265**		
SOG.R	**0.0004/8.8791**	**0.0012/−3.4971**		
MOG.L	**0.0015/7.2828**	**0.0017/−3.3755**		
MOG.R	**0.0010/7.8302**	**0.0003/−3.9269**		
SMG.L	0.0197/4.1984	**0.0009/−3.5994**		
HES.L	0.006/5.5912	**0.002/−3.3075**		
HES.R	0.028/3.8009	**0.0063/−2.8904**		

*p-value < 0.01, false discovery rate (FDR) corrected. Brain regions with the p < 0.01 are shown in bold. iRBD, idiopathic rapid eye movement sleep behavior disorder; PD, Parkinson’s disease; HC, healthy control.*

**TABLE 3:** Alterations of Nodal Efficiency among idiopathic rapid eye movement sleep behavior disorder (iRBD), Parkinson’s disease (PD), and healthy control (HC).

Brain regions	*P/T* (RBD vs. PD vs. HC)	*P/T* (PD vs. HC)	*P/T* (RBD vs. HC)	*P/T* (RBD vs. PD)
ROL.L	0.0063/5.5255	**0.0021/−3.2961**	0.0164/−2.5154	0.7735/0.2899
ROL.R	0.0083/5.1959	**0.0023/**−**3.2586**	0.1025/−1.6744	0.2537/1.1590
OLF.R	**0.0048/5.8544**	**0.0019/**−**3.3285**	0.0500/−2.0264	0.3051/1.0395
ORBsupmed.R	**0.0033/6.3056**	**0.0003/**−**3.9984**	0.0304/−2.251	0.3383/0.9698
INS.L	**0.0041/6.0523**	**0.0026/**−**3.2209**	0.0067/−2.8749	0.7591/0.3089
INS.R	**0.0011/7.6270**	**0.0009/**−**3.5936**	0.0047/−3.0126	0.3821/0.8842
ACG.L	**0.0007/8.2539**	**0.0002/**−**4.0450**	0.0339/−2.2035	0.0736/1.8402
ACG.R	**0.0003/9.2689**	**0.0002/**−**4.1187**	0.0062/−2.9055	0.1245/1.5707
MCG.L	0.0069/5.4149	**0.001/**−**3.5698**	0.0970/−1.7026	0.2505/1.1671
MCG.R	0.0057/5.6497	**0.0016/**−**3.3951**	0.0140/−2.5807	0.6008/0.5276
CAL.L	**0.0029/6.4614**	**0.0013/**−**3.458**	0.0259/−2.3209	0.5394/0.6193
CAL.R	**0.0003/9.5218**	**0.0000/**−**4.6155**	0.0130/−2.6089	0.3176/1.0127
CUN.L	**0.0017/7.1434**	**0.0012/**−**3.4843**	0.0599/−1.9412	0.1514/1.4639
CUN.R	**0.0003/9.4948**	**0.0002/**−**4.1101**	0.0086/−2.7738	0.1863/1.346
LING.L	**0.0008/8.0600**	**0.0020/**−**3.3097**	**0.0006/**−**3.773**	0.8507/−0.1895
LING.R	**0.0018/7.0711**	**0.0019/**−**3.3265**	0.0074/−2.8348	0.6396/0.4721
SOG.L	**0.0003/9.2967**	**0.0003/**−**3.9737**	**0.0006/**−**3.7381**	0.7705/0.2939
SOG.R	**0.0001/11.2032**	**0.0002/**−**4.078**	**0.0003/**−**3.9751**	0.7689/0.296
MOG.L	**0.0012/7.5181**	**0.0009/**−**3.5889**	0.0031/−3.1673	0.8966/0.1308
MOG.R	**0.0001/10.5401**	**0.0001/**−**4.5319**	**0.0010/**−**3.5647**	0.6204/0.4993
FFG.L	0.0148/4.5288	**0.0037/**−**3.0904**	0.0408/−2.12	0.5721/0.5699
FFG.R	0.0072/5.3743	**0.0040/**−**3.0623**	0.0109/−2.6802	0.7239/0.3558
PoCG.R	0.0106/4.9186	**0.0045/**−**3.0159**	0.0257/−2.3241	0.4842/0.7065
SMG.L	**0.0023/6.7315**	**0.0002/**−**4.1433**	0.0241/−2.3525	0.4033/0.8451
SMG.R	0.0056/5.6765	**0.0007/**−**3.6584**	0.0097/−2.7287	0.5479/0.6064
HES.L	**0.0019/6.9827**	**0.0007/**−**3.6999**	0.0194/−2.4445	0.2137/1.2646
HES.R	0.0157/4.4594	**0.0035/**−**3.1138**	0.1763/−1.3785	0.1938/1.3228
TPOmid.L	0.0104/4.934	**0.0012/**−**3.4946**	0.0804/−1.7977	0.3594/0.9277

*p-value < 0.005, FDR corrected. Brain regions with the p < 0.005 are shown in bold. iRBD, idiopathic rapid eye movement sleep behavior disorder; PD, Parkinson’s disease; HC, healthy control.*

**TABLE 4:** Alterations of Nodal Shortest Path among iRBD, PD, and HC.

Brain regions	P/T (RBD vs. PD vs. HC)	P/T (PD vs. HC)	P/T (RBD vs. HC)	P/T (RBD vs. PD)
PreCG.L	0.1483/1.9713	0.0861/1.7611	**0.0028/**−**3.1964**	0.5715/0.5708
PreCG.R	0.0079/5.2590	0.0882/1.749	**0.0013/**−**3.4878**	0.1166/1.6057
ROL.R	1.0000/0.0000	1.0000/0.0000	**0.0010/**−**3.5683**	1.0000/0.0000
SMA.R	0.5272/0.6471	0.3511/−0.9438	**0.0038/**−**3.0854**	0.5463/0.6087
REC.R	1.0000/0.0000	1.0000/0.0000	**0.0036/**−**3.1071**	1.0000/0.0000
ACG.L	0.0095/5.0484	**0.0027/3.1975**	0.0092/−2.7492	0.1367/−1.5203
ACG.R	0.0051/5.7868	**0.0017/3.3619**	0.0693/−1.8708	0.1211/−1.5855
MCG.L	0.1987/1.6611	0.0881/1.7495	**0.0009/**−**3.5923**	0.6056/−0.5207
CAL.L	0.0050/5.7970	**0.0012/3.4888**	0.0422/−2.1047	0.6320/−0.4827
CAL.R	**0.0008/8.1311**	**0.0001/4.4219**	0.0781/−1.8122	0.5094/−0.6661
LING.L	**0.0012/7.5722**	**0.0014/3.4467**	0.1236/−1.5759	0.8790/0.1533
LING.R	1.0000/0.0000	**0.0006/3.7305**	0.0277/−2.2913	1.0000/0.0000
SOG.L	1.0000/0.0000	**0.0002/4.0386**	0.1529/−1.4594	1.0000/0.0000
SOG.R	**0.0004/9.0928**	**0.0002/4.1652**	0.2118/−1.2705	0.9537/−0.0584
PoCG.R	1.0000/0.0000	1.0000/0.0000	**0.0025/**−**3.2423**	1.0000/0.0000
SMG.L	1.0000/0.0000	1.0000/0.0000	**0.0050/**−**2.9888**	1.0000/0.0000
STG.L	1.0000/0.0000	0.0392/2.1339	**0.0025/**−**3.2492**	1.0000/0.0000
STG.R	1.0000/0.0000	1.0000/0.0000	**0.0005/**−**3.8196**	1.0000/0.0000

*p-value < 0.005, FDR corrected. Brain regions with the p < 0.005 are shown in bold. iRBD, idiopathic rapid eye movement sleep behavior disorder; PD, Parkinson’s disease; HC, healthy control.*

### Correlations Between Global and Local Topological Properties and Cognition in Idiopathic Rapid Eye Movement Sleep Behavior Disorder and nRBD-PD Group

**FIGURE 3:**
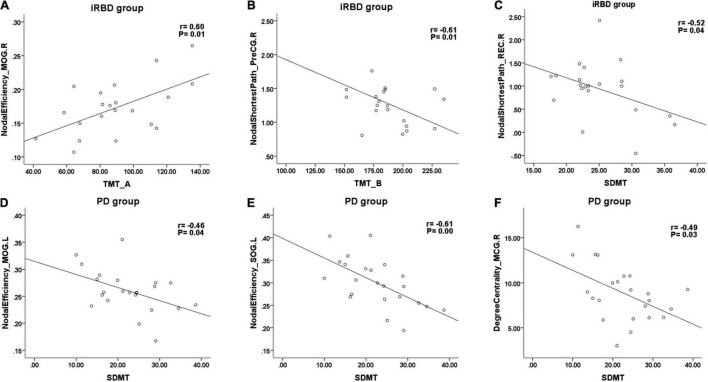
Representative scatterplots between cognitive tests and local properties in iRBD and PD. SDMT, symbol digit modalities test; TMT-A, part A of trail making test; TMT-B, part B of trail making test; PreCG, precentral gyrus; REC, rectus gyrus; MOG, middle occipital gyrus; SOG, superior occipital gyrus; MCG, middle cingulate gyrus.

Cognitive tests and global/local properties that differed with the HC group were chosen for further partial correlation analysis in both iRBD and nRBD-PD groups. In the iRBD group, after adjusting for age, gender, and education, the TMT-A score was positively correlated with the nodal efficiency of the middle occipital gyrus (*r* = 0.604, *p* = 0.013) (Figure 3A). SDMT score was negatively correlated with the nodal shortest path length of rectus gyrus and precentral gyrus (*r* = −0.52, −0.515; *p* = 0.038, 0.041, respectively) (Figure 3C). TMT-B score was negatively correlated with the nodal shortest path length of precentral gyrus and the superior temporal gyrus (*r* = −0.607, −0.555; *p* = 0.013, 0.026, respectively) (Figure 3B).

In nRBD-PD, SDMT score was negatively correlated with the degree centrality of the middle cingulate gyrus (*r* = −0.487, *p* = 0.029) (Figure 3F) and negatively correlated with the nodal efficiency of superior occipital gyrus, cuneus, middle occipital gyrus, and middle cingulate gyrus (Figures 3D,E). Lastly, the TMT-A score was positively correlated with the nodal efficiency of the right middle occipital gyrus.

## Discussion

The present study revealed attention, executive, and some memory deficits in patients with iRBD and early-stage nRBD-PD. Most of these deficits were serious in nRBD-PD. Graph theory analysis identified small-worldness was still present in iRBD and early-stage nRBD-PD. However, at the global level, both iRBD and early-stage nRBD-PD showed reduced global and local efficiency and increased characteristic path length, which were similar to previous studies (Luo et al., 2015; Sreenivasan et al., 2019; Li et al., 2020; De Micco et al., 2021).

At the local level, compared with the HC group, the nodal efficiency and degree centrality were decreased, and the nodal shortest path was increased in the nRBD-PD group. The altered brain regions were most extensive on nodal efficiency. By numbers of altered brain regions, the sensitivity of nodal properties for nRBD-PD network changes was in the order of nodal efficiency, degree centrality, and nodal shortest path. Similarly, in the comparisons of local properties between iRBD and HC, the parameter of nodal efficiency also depicted the most altered brain regions. On nodal efficiency, nRBD-PD exhibited more affected brain regions than iRBD. The affected brain regions in iRBD were largely included in the regions of nRBD-PD.

The brain regions with decreased nodal efficiency in both nRBD-PD and iRBD groups contained the occipital regions. In the comparison of the three groups, only the occipital lobe showed abnormalities in nodal efficiency, degree centrality, and nodal shortest path. The occipital lobe may be vulnerable to synucleinopathies. Several neuroimaging studies have found that the occipital lobe was structurally and functionally damaged in iRBD. The atrophy of the lingual gyrus and fusiform gyrus were seen on structural neuroimaging (Rahayel et al., 2015; Campabadal et al., 2019). Functional imaging revealed decreased occipital perfusion and metabolism in patients iRBD and iRBD with concomitant mild cognitive impairment (iRBD-MCI). Hypometabolism of the occipital-parietal lobe on ^18^fluorodeoxyglucose (FDG) PET scan (FDG-PET) was an important part of iRBD-related pattern (iRBDRP) (Wu et al., 2014). In addition, occipital damage may be related to the conversion of RBD to Dementia with Lewy Bodies (DLB) (Ota et al., 2016). Cortical damage in iRBD was discussed in detail in recent review articles of RBD neuroimaging (Ferini-Strambi et al., 2019; Campabadal et al., 2021).

Interestingly, for the nodal shortest path, there were opposite changes in the iRBD group vs. the nRBD-PD group. First, patients with iRBD showed decreased nodal shortest path length, whereas the nRBD-PD group showed the increased nodal shortest path. The decreased shortest path may be due to the formation of a new direct path or the enhancement of the original path. Studies have found that the volume of hippocampus, cerebellum, and striatum increased in iRBD, suggesting early compensation (Campabadal et al., 2021). Second, the altered brain regions in the iRBD group were mainly located in the temporal lobe, while the nRBD-PD were mainly located in the occipital lobe. This implies that iRBD has its own unique characteristics compared with nRBD-PD. Indeed, the future phenoconversion of iRBD is not just PD, but also DLB and MSA.

Several longitudinal studies have identified baseline attention/executive deficits as risk factors for future phenoconversion in iRBD, particularly conversion to DLB (Ferini-Strambi et al., 2019). The present study found both nRBD-PD and iRBD presented some degree of impairment in attention and executive functions. In the iRBD group, the TMT-A score was positively correlated with the nodal efficiency in the middle occipital gyrus. This implied that occipital lobe damage was involved in attention deficits in iRBD. It was well established that the occipital lobe was widely connected with the intraparietal sulcus and the frontal eye fields to form the dorsal attention network (DAN) (Vossel et al., 2014; Suo et al., 2021). TMT-B and SDMT scores in the iRBD group were negatively correlated with local properties of frontotemporal regions, suggesting that the executive function of iRBD may be interfered by these nodes or their connections. In the nRBD-PD group, the SDMT score was mainly negatively correlated with the nodal efficiency of occipital regions. In a meta-analysis of correlation studies between the fMRI and SDMT test, SDMT is mainly related to the frontoparietal attentional network, occipital cortex, cuneus, precuneus, and cerebellum (Silva et al., 2018). The SDMT test used in this study was the written version which requires the participation of the visual cortex.

Compared with the HC group, we did not find decreased AVLT short- and long-DR scores in the RBD group, which was different from some studies. Recently, one detailed study on cognitive impairment in iRBD and PD found that iRBD performed better in long-term memory, presumably due to compensation in the early stage (Assogna et al., 2021). Previous studies have found that the volume of hippocampus and parahippocampal gyrus and hippocampal perfusion increased in RBD (Mazza et al., 2006; Scherfler et al., 2011).

The main strength of our study lies in the direct comparison of iRBD and early-stage nRBD-PD. Previous studies were mostly conducted in patients with PD with and without RBD. The weakness of the present study is the small sample size and cross-sectional design. In this study, to avoid visual interference, all subjects had their eyes closed during scanning. Subjects were instructed not to fall asleep before examination and were inquired whether they fell asleep during scanning after the examination. Even this, we could not ensure the awake state of all subjects during the resting state scan without fMRI-compatible electroencephalogram (EEG).

In conclusion, both global and local efficiency of functional networks declined in the nRBD-PD and iRBD groups. The overlaps and differences in local topological properties between nRBD-PD and iRBD indicate that iRBD not only shares functional changes of PD but also presents distinct features. It is worth exploring the local topological properties as the imaging markers to predict future cognitive deterioration in iRBD.

## Data Availability Statement

The raw data supporting the conclusions of this article will be made available by the authors, without undue reservation.

## Ethics Statement

The studies involving human participants were reviewed and approved by the Medical Ethics Committee of People’s Hospital of Zhengzhou University. The patients/participants provided their written informed consent to participate in this study.

## Author Contributions

All authors listed have made a substantial, direct, and intellectual contribution to the work, and approved it for publication.

## Conflict of Interest

The authors declare that the research was conducted in the absence of any commercial or financial relationships that could be construed as a potential conflict of interest.

## Publisher’s Note

All claims expressed in this article are solely those of the authors and do not necessarily represent those of their affiliated organizations, or those of the publisher, the editors and the reviewers. Any product that may be evaluated in this article, or claim that may be made by its manufacturer, is not guaranteed or endorsed by the publisher.

## Footnotes

1
http://www.restfmri.net/

2
http://www.nitrc.org/projects/gretna/
